# Metformin Therapy for Acne Vulgaris: A Meta-Analysis

**DOI:** 10.3390/ph17060728

**Published:** 2024-06-05

**Authors:** Lidia Szefler, Weronika Szybiak-Skora, Anna Sadowska-Przytocka, Ryszard Zaba, Barbara Wieckowska, Katarzyna Lacka

**Affiliations:** 1 Students’ Scientific Society at Poznań University of Medical Sciences, Student’s Scientific Section of Endocrinology, Department of Endocrinology, Metabolism and Internal Medicine, Poznań University of Medical Sciences, 61-701 Poznan, Poland; szefler.lidia@gmail.com (L.S.); weronikaszybiak@gmail.com (W.S.-S.); 2 Department of Allergic and Occupational Skin Diseases, Poznań University of Medical Sciences, 61-701 Poznan, Poland; 3 Department of Dermatology and Venerology, Poznań University of Medical Sciences, 61-701 Poznan, Poland; ryszard.zaba@usk.poznan.pl; 4 Department of Computer Sciences and Statistics, Poznań University of Medical Sciences, 61-701 Poznan, Poland; barbara.wieckowska@ump.edu.pl; 5 Department of Endocrinology, Metabolism and Internal Medicine, Poznań University of Medical Science, 61-701 Poznan, Poland; kktlacka@ump.edu.pl

**Keywords:** acne vulgaris, insulin resistance, metformin treatment, meta-analysis

## Abstract

Acne vulgaris is a common disease, which occurs in adolescents as well as adults and has a significant influence on the patient’s quality of life (QoL) in every aspect. Due to resistance to standard therapies, it has become necessary to prospect for new treatment strategies. It is important to highlight that the diagnosis and treatment of the underlying cause of acne such as metabolic and hormonal disorders may significantly improve the effectiveness of acne treatment. The correlation between Insulin Resistance (IR) and acne has been proven. Both disorders share many common occurrence factors and activation pathways. Metformin, an antihyperglycemic agent, seems to be a possible therapy option, not only because of its insulin sensitizing ability but also via plenty of additional effects of this medicine. While the efficiency of metformin therapy in patients with acne and Polycystic Ovary Syndrome (PCOS) is well explored, it is still necessary to evaluate it in patients without any endocrinopathies. This meta-analysis aimed to estimate the effectiveness of oral metformin as a monotherapy in acne patients without PCOS or other endocrinopathies. Study selection was performed with included criteria such as no PCOS and other endocrinopathies diagnosed, oral administration of metformin, and metformin in monotherapy. Selected studies contained comparisons in the Global Acne Grading System (GAGS) before and after metformin therapy. Statistical analysis detected significant improvement in skin condition after treatment with metformin.

## 1. Introduction

Acne is one of the most common dermatological diseases. It mostly affects adolescents (more than 95% of boys and 85% of girls); moreover, 50% of them continue to present with acne in adulthood [1]. Acne may be triggered by many modifiable and nonmodifiable factors such as hormonal and metabolic disorders, genetic predispositions, unhealthy diet, smoking, stress, seasonal factors, incorrect skin care, and many others. Adolescent acne and adult acne significantly differ from each other in gender, localization, lesion types, response to treatment, and tendency to scarring. Adolescent acne more often affects males, while females often suffer from adult acne. Depending on the moment, when acne appears for the first time, we can define it as “persistent acne”, when it has started in adolescents, or “late-onset acne”, when it occurs for the first time after the age of 25 [2]. Typical places of acne occurrence are the neck, face, upper chest, back, and shoulders, where there is a high density of sebaceous glands. Acne is a complex disease with four main factors: intensified sebum secretion, abnormal keratinization, skin colonization by Propionibacterium acnes, and inflammation [3,4,5]. In general, acne lesion type might be divided into two groups—inflammatory and non-inflammatory. Non-inflammatory lesions contain closed or opened comedones, and inflammatory contain nodules, pustules, papules, and cysts. Acne vulgaris may be defined as mild, moderate, or severe depending on the severity and type of lesions [6].

This disease has a negative influence on many areas of a patient’s life, impacts social and psychological aspects, and, as a result, worsens the patient’s quality of life (QoL) [1]. Acne may cause various psychological problems and diseases in patients of all ages and genders. Patients with acne vulgaris have significantly low self-esteem and self-confidence, which may lead to anxiety and depression and increase the suicidal risk [7]. Moreover, disturbed emotional states in patients with acne affect every sphere of their lives, and as a result, it worsens the patient’s vocational, social, and academic functioning [8]. The impact on a patient’s life seems to be comparable to other systemic diseases [9]. Considering the negative consequences for a patient’s life, it is extremely important to prospect for effective and safe treatment methods which allow treating patients successfully and without serious side effects of acne or treatment.

The current guidelines for caring for patients with acne vulgaris are based on topical and systemic therapies and physical modalities. All of the therapies contain various substances and methods. Antibiotics, hormonal agents, and isotretinoin are used as a foundation of systemic treatment. The choice of treatment strategies depends on the type and severity of the disease as well as its location [10]. Due to limitations such as resistance to recommended therapies and multiple side effects, there is still a need to prospect for new treatment strategies [11,12]. While there is plenty of evidence supporting the effectiveness of using metformin to treat acne in patients with Polycystic Ovary Syndrome (PCOS), it is still necessary to analyze its usefulness in treating acne in patients who do not have any underlying endocrine disorders [13,14,15,16].

The latest outcomes prove the correlation between Insulin Resistance (IR) and acne vulgaris not only in female patients with PCOS but also in men and patients without a PCOS diagnosis [17]. Acne and IR seem to have similar underlying pathomechanisms. An increase in the serum level of insulin induces a decrease in insulin-like growth factor binding protein (IGF-binding protein), and as a result, it raises the free serum level of insulin-like growth factor 1 (IGF-1). Altered glucose metabolism also causes androgen secretion stimulation as a consequence of insulin action, as well as lowering the serum level of sex hormone binding globulin (SHBG). The mentioned abnormalities encourage severe mammalian target of rapamycin complex 1 mTORC1 activity which was detected in patients with acne [18].

Metformin is an antihyperglycemic agent recommended for those with type 2 diabetes [19]. Moreover, in recent years it has been an object of interest for potential use in treating other conditions such as inflammatory and non-inflammatory skin diseases. The exact mechanisms of metformin action in dermatological diseases are not yet well understood. Metformin is applicable in Hidradenitis Suppurativa and causes a reduction in androgens levels by inhibiting their overproduction. Via a reduction in the expression of melanogenic proteins, it also may be used in the cure of acanthosis nigricans and melasma. Metformin also decreases inflammation by lowering inflammatory cytokines and for this reason, can be used in patients with psoriasis or allergic contact dermatitis. Another mechanism that can be used in treating skin disorders is its antiproliferative effect; thus, it could be an option for different skin cancers [20,21,22,23,24,25]. The major mechanism of lowering blood sugar levels by metformin is decreasing the hepatic glucose output and encouraging muscles and adipocytes to glucose utilization, which has a positive impact on insulin sensitivity. Metformin also causes an inhibition of mTORC1 via activity in the AMP-activated protein kinase (AMPK) pathway. Furthermore, it reduces the serum level of IGF-1 and may reduce inflammation [26]. It has been also discovered that metformin influences lipid metabolism, mostly decreasing triglyceride and free fatty acid. This medication seems to have an abundance of additional effects, which may be used in mentioned skin diseases, including acne [27].

Besides metformin’s ability to improve acne intensity by regulating insulin metabolism, there is also the possibility that it affects the gut microbiome, which seems to have a relationship with the severity of acne [28].

This meta-analysis aimed to estimate the efficiency of the use of oral metformin as monotherapy in patients with acne but without PCOS or other endocrinopathies.

## 2. Results

We selected three articles to analyze containing data that were connected with metformin treatment and the clinical presentation of acne vulgaris. We decided to compare groups of patients taking into account age, body mass index (BMI), sex, methods of treatment, and the differences between the Global Acne Grading System (GAGS) before and after treatment. Studies included in this meta-analysis concerned metformin treatment in patients with acne that was unrelated to PCOS or other endocrinopathies. In all of the articles considered, patients were treated only with metformin, without any other medicines. Compiled data are presented in Table 1.

**Figure 1:**
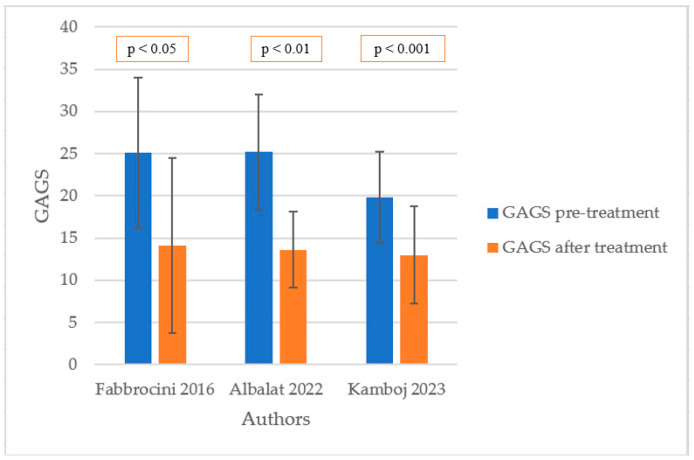
GAGS before and after treatment in selected studies [29,30,31].

The listed articles contained sufficient data on the effect of metformin therapy on GAGS in patients with acne vulgaris. All of the selected studies demonstrated clinical improvements in skin conditions after treatment. The differences between pre-treatment and after-treatment GAGS in each study are presented in Figure 1.

**Figure 2:**
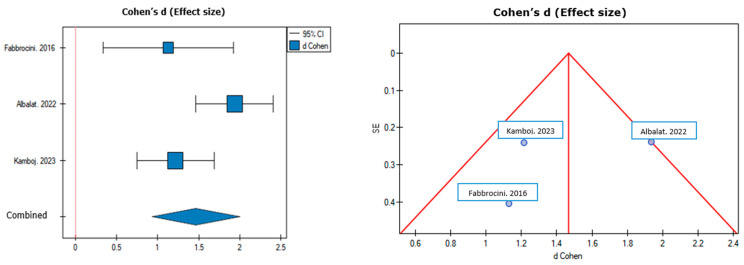
Cohen’s d effect size with 95% CI [29,30,31].

The heterogeneity between studies was I^2^ = 63.79% (95% CI for I^2^ = 0% to 89.64%; *p* = 0.063). The results of Cohen’s d show that there is a statistically significant publication load. Cohen’s d = 1.47 (SE = 0.27; 95% CI = 0.93 to 2.00; *p* = 0.00000008). The results of our meta-analysis are presented in Table 2 and Figure 2.

The results of our meta-analysis proved a statistically significant (*p* = 0.00000008) improvement in GAGS after metformin treatment in patients with acne vulgaris.

## 3. Discussion

Acne vulgaris is a disease that affects a large part of society of all ages and genders. Because of the significant influence on a patient’s QoL, it is necessary to discover the most appropriate way to treat acne, increase the effectiveness of treatment, and reduce the side effects of both acne and the treatment itself [1,9].

In recent years, acne vulgaris treatment methods have started to change. Symptomatic therapies turned out to be insufficient and there was a need to find and treat the causes of acne. Possible connections between acne and other diseases or abnormalities such as IR, PCOS, hyperandrogenism, and other hormonal disorders were studied [13,17,32,33,34]. 

Our study focuses on evaluating the efficiency of metformin in patients without symptoms other than IR endocrinopathy. A connection between acne and PCOS occurrences has been well investigated. PCOS is associated with plenty of hormonal disorders, including IR [13,14,15,16]. It was also meaningful to indicate the relationship between IR as the only presented hormonal disorder and acne [17,18,35]. Due to the availability of many studies on the use of metformin in patients with PCOS, we decided to consider its usefulness in patients without this endocrinopathy. It is the first meta-analysis of metformin treatment in acne with the presence of PCOS as an excluded criterion. 

The altered hormonal profile included increased insulin and IGF-1 was observed in patients with acne [36]. There is some evidence that IR might be a factor in acne occurrence. The IR may coexist with increased glucose fasting serum level, but it is not necessary. To diagnose this irregularity, we need to calculate the Homeostatic Model Assessment of Insulin Resistance (HOMA-IR), which is based on a formula containing the levels of insulin and glucose fasting serum levels [37]. Acne vulgaris and IR seem to have similar mechanisms of development and the relationship between them has been noticed [17,18,35,38,39,40,41,42,43]. Western diet characterized by a high glycemic load and being rich in dairy products also causes overstimulation of the mTORC1 activity pathway and may cause acne [44,45]. One of the factors activating the mTORC1 pathway is leucine. Vegan or vegetarian diets contain decreased levels of leucine compared to diets with meat and fish. No-meat diets seem to have an influence on skin conditions and the improvement in acne lesions [46]. Moreover, gut microbiomes and their metabolites also have an impact on the mTORC1 pathway. Some studies proved the positive influence of probiotics curation consisting of Lactobacillus rhamnosus GG on inflammatory processes, the reduction in IGF1 levels, and the improvement in acne lesions. Probiotics seem to be a subsequent part of acne treatment in the future, but more studies conducted on larger and appropriate populations are still necessary [47]. The diagnosis of IR in patients with resistant acne to common treatment could turn our attention to the underlying cause of acne and change therapy methods to be more appropriate. 

Moreover, BMI status and family history are also considered factors that influence acne presentation. Heng et al. [48] showed a statistically significant influence of overweight and obese BMI results on acne presentation compared to normal range/underweight BMI (OR of 2.36; 95% CI 1.97–2.83; overweight/obese BMI with reference to normal/underweight BMI). Patients with excessive amounts of adipose tissue present increased levels of adipokines and inflammatory mediators, which intensify skin inflammatory changes. One of the most important adipokines is leptin, in which the expression is promoted in response to ductal hipoxia in follicles and hypoxia-inducible factor 1-α (HIF-1α). The activity of mTORC1 is also correlated with HIF-1α promotion. Metformin inhibits mTORC1 activity via its influence on the AMPK pathway and reduces inflammation [49].

Fabbrocini et al. [29] engaged 20 male patients with altered metabolic profiles in the study, with 10 as a study group and 10 as a control. The study group was treated with metformin 500 mg twice daily and a hypocaloric diet for 6 months. No patients reported any side effects. Results show a significant reduction in the GAGS in the metformin group when there was no statistically important difference in the control. GAGS before therapy was 25.1 (±8.9) and after was 14.1 (±10.4). A correlation between severe acne (GAGS) and disturbed metabolic parameters (BMI, waist-to-hip ratio, HOMA-IR) was also detected. 

Albalat et al. [30] performed a study on 50 patients with resistant acne to other treatment methods during the previous 6 months. Patients received 500 mg of metformin twice daily for 4 months. Parameters such as BMI, GAGS, and serum level of IGF-1 were recorded before and after metformin therapy. Six patients experienced mild gastrointestinal upset (nausea and vomiting) and three mild hypoglycemia episodes. There was a significant decrease in all collected variables among the studied group after treatment compared to the condition before. GAGS before therapy was 25.2 (±6.8) and after was 13.6 (±4.5).

A different study was performed by Kamboj et al. [31] on 30 patients with acne vulgaris with the use of metformin 1000 mg daily for 3 months without any other systemic or topical therapies. None of the patients experienced any side effects associated with metformin therapy. As a result, acne grading presented as GAGS decreased significantly compared to the state before treatment. GAGS before therapy was 19.8 (±5.4) and after was 13 (±5.79). However, unlike most articles, this study shows an increase in IGF-1 serum levels, which is surprising. Many other parameters were examined in this study, but this is not an object of interest in our study. There are a few more papers about the efficacy of metformin use in acne treatment not included in our meta-analysis. It was caused by a method of metformin administration as an adjuvant therapy and not in monotherapy as happened in included studies. Nevertheless, it is not less important to notice the usefulness of metformin as an additional medicine to other systemic treatments. 

Robinson et al. [50] accomplished a study on 76 patients with moderate to severe acne, with 39 in the study group and 37 in control for 12 weeks. The study group received topical benzoyl peroxide 2.5%, tetracycline 250 mg twice daily, and metformin 850 mg daily; the control group received treatment without metformin. Patient examination was based on the evaluation of the severity of acne by measuring lesion count, inflammatory lesion counts, non-inflammatory lesion counts, Investigator’s Global Assessment grading (IGA), and Cardiff Acne Disability Index (CADI). Also, estimated BMI and blood samples to evaluate hepatic and kidney function and glucose metabolism were taken. As many as 31.7% of the patients receiving metformin reported mild and temporary gastrointestinal side effects. The authors suggested that changes in the way of metformin administration, such as using the sustained release variety or gradually increasing the dose, may reduce the patients’ experience of side effects. In all evaluation measurements, the metformin group showed better outcomes than the control.

Gabaton et al. [51] investigated the effectiveness of metformin as an adjunct to lymecycline and topical treatment. Forty patients with moderate to severe acne received metformin or placebo tablets with lymecycline and topical therapy (benzoyl peroxide gel). The antibiotic was received for 6 weeks and other medicines for 18 weeks. The evaluation was performed every two weeks by checking the reduction in total inflammatory and non-inflammatory lesion count, Dermatology Life Quality Index Score (DLQI), and subjective self-assessment score. As a result, metformin turned out to be a useful and safe adjunct therapy to lymecycline.

Sadati et al. [52] compared the effectiveness of metformin vs. doxycycline. Forty patients were randomly divided into two groups; the first one was receiving oral metformin 500 mg twice daily, and the second one received doxycycline 100 mg once daily for 2 months. Both groups also received recommendations to use 5% benzoyl peroxide every night and to use Sun Protection Factor (SPF) daily. Patients were followed up on and examined every month. Results were estimated using GAGS, IGA, CADI, Total Lesions Count (TLC), and the number of inflammatory and non-inflammatory lesions. Both therapies proved to be comparably safe; three patients in the metformin group were affected by gastrointestinal upset (described as mild to moderate), and one patient in the doxycycline group reported mild photosensitivity. This study shows similar improvements in the severity of acne after treatment with metformin or doxycycline. However, doxycycline was more effective in decreasing the number of inflammatory lesions.

Another study conducted by Deng et al. [28] evidenced a correlation between the composition of gut microbiota and the severity of acne and the possibility of modifying this dysfunction with metformin therapy. Patients aged 18–30 were allocated to two groups; one received only isotretinoin 0.25 mg/kg/d, and the second one isotretinoin with metformin 500 mg daily for 12 weeks. Patients with comorbidities, who were addicted to smoking or alcohol, and those who were not treatment-naive were excluded from the study. Acne grading after treatment was lower in the metformin group compared to the isotretinoin-only group.

It is also important to consider the safety of metformin therapy. In the mentioned studies, patients experienced only mild side effects, and it did not cause the discontinuation of the study. They mostly reported gastrointestinal upset and, less often, hypoglycemia episodes. There are plenty of other side effects that metformin may cause. However, patients with acne in cited studies have not experienced any of them. Other possible metformin side effects recognized as common (despite mentioned gastrointestinal symptoms and hypoglycemia episodes) are decreased appetite, altered taste, and vitamin B12 deficiency [20]. 

Metformin is excreted from the body mainly by tubular secretion in the kidneys. A percentage of 90% of metformin taken orally is excreted by the kidneys within 24 h after ingestion [53]. Therefore, renal dysfunction is a limitation of the use of metformin. Treatment with the use of metformin could be initiated given an estimated glomerular filtration rate (eGFR) of 45 to 60 mL/min. This treatment can be continued in patients with an eGFR of 30 to 45 mL/min with an examination of kidney function. The contraindication to continue metformin therapy is an eGFR value of less than 30 mL/min [54,55]. The toxicity of metformin is still a subject of study. Previous animal studies have shown toxicity at the following doses depending on how the drug was taken:

- Oral LD50 (rat): 1 g/kg;
- Intraperitoneal LD50 (rat): 500 mg/kg;
- Subcutaneous LD50 (rat): 300 mg/kg;
- Oral LD50 (mouse): 1450 mg/kg;
- Intraperitoneal LD50 (mouse): 420 mg/kg;
- Subcutaneous LD50 (mouse): 225 mg/kg [56,57].

The human strict toxic dose that leads to metabolic acidosis and hyperlactatemia is still unclear. Patients who received a dose ranging from 3.5 g to 22.5 g did not develop severe metabolic complications [58,59]. As for the group of pediatric patients, Lacher described the case of a 15-year-old girl who was admitted at a 38.25 g dosage as being complicated by lactic acidosis and renal dysfunction [60]. To summarize previous studies, more data are needed to establish the LD50 and exact toxicity threshold for metformin.

Common acne treatments contain topical and systemic therapies such as topical retinoids, benzoyl peroxide, topical antibiotics, salicylic/azelaic acids, aluminum chloride, zinc, sulfur and resorcinol, systemic antibiotics (tetracyclines, macrolides, clindamycin, trimethoprim, and ampicillin/amoxicillin), hormonal agents (contraceptive pill, antiandrogens, spironolactone, and oral corticosteroids), and isotretinoin. One of the therapeutic possibilities is also physical modalities and alternative therapies. Thus, there are plenty of diverse options for acne treatment, which can be used in various combinations [10,61].

Unfortunately, many of them may cause unfavorable side effects for patients. Oral antibiotics are mostly administered to patients with moderate to severe acne. Possible side effects depend on the group of antibiotics. Tetracyclines disturb the expansion of the teeth and bones and should not be administered to children under the age of 8 [10,62]. They may also induce gastrointestinal upset, undesirable skin reactions, photosensitivity, and vaginal candidiasis. Macrolides mostly provoke gastrointestinal symptoms, and they may affect other drugs metabolism. Oral contraceptive pills are usually well tolerated because most adverse effects disappear after three cycles of taking these pills. The most serious side effects of spironolactone are hyperkalemia, hypotension, and feminization of the male fetus (when taken prenatally) [63]. Isotretinoin is used in patients in whom other forms of treatments turned out to be ineffective. Nevertheless, administering it to female patients of childbearing age is associated with a high risk of teratogenicity. Most patients receiving isotretinoin are affected by mucocutaneous side effects. Hair loss, hepatitis, and disturbed lipid metabolism also may be isotretinoin-related [63,64]. A new drug from the retinoid group with a much better safety profile is Trifarotene. It is a new fourth-generation retinoid with a selective effect on RAR-γ. Due to the selected action, the number of side effects associated with the use of RAR-β has been reduced [65].

Considering the serious side effects of some of the therapies, metformin seems to have a promising safety profile. Thus, it could be a therapeutic option for patients for whom the side effects of other treatments are unbearable and unacceptable.

The effective use of hormonal agents in common acne treatment gives hope that other hormonal drugs, such as metformin, may also be useful in acne treatment.

Our study presents numerous limitations. The main limitation is the small number of studies in which metformin was used as the single therapy for patients with acne vulgaris. We also excluded many studies due to the presence of additional diseases or chronic treatment in the study groups. Another problem is the duration of use and dosage of metformin, which differs between individual studies. Individual studies also refer to different age groups, which also makes it difficult to compare the effectiveness of treatment. Being aware of the numerous limitations, we would like to emphasize the importance of the results obtained on the basis of selected studies, encourage standardized studies on a larger group of patients, and open a clinical discussion on the effectiveness and safety of metformin as monotherapy in patients with acne vulgaris.

## 4. Materials and Methods

### 4.1. Database Research

The research was performed according to Preferred Reporting Items for Systematic Reviews and Meta-Analyses (PRISMA). The search was conducted by two independent people. Below are enlisted databases that were used: PubMed, Google Scholar, Scopus, and Medline. The mentioned databases are a proper source for acquiring medical knowledge. The following phrases were used to conduct the research: “Acne Vulgaris”, ”Metformin and Acne”, “Biguanides and Acne”, “Metformin Therapy in Patients with Acne”, and ”Insulin Resistance and Acne”. The investigation was also executed without a time limit, starting in November 2023. The country of origin was insignificant.

### 4.2. Study Selection

This study aimed to evaluate the efficiency of the use of metformin in patients with acne without PCOS. Included articles contain results about the severity of acne and other parameters before and after metformin treatment in patients with acne. Depending on the papers, patients were treated only with metformin or with metformin as an adjuvant therapy to the systemic or topical medications, but finally, articles with only metformin-treated patients were included in the meta-analysis. The main excluded criterion was the occurrence of PCOS. The search was accomplished after careful consideration of all the relevant factors, such as clinical group, control group, accompanying diseases, and metformin dosage method. The inclusion criteria that were used are set out in order of mention below:

- No PCOS and other endocrinopathies diagnosed;
- Oral administration of metformin;
- Metformin in monotherapy;
- Articles allowing the determination of the Cohen’s d effect size;
- Full-text version available.

**Figure 3:**
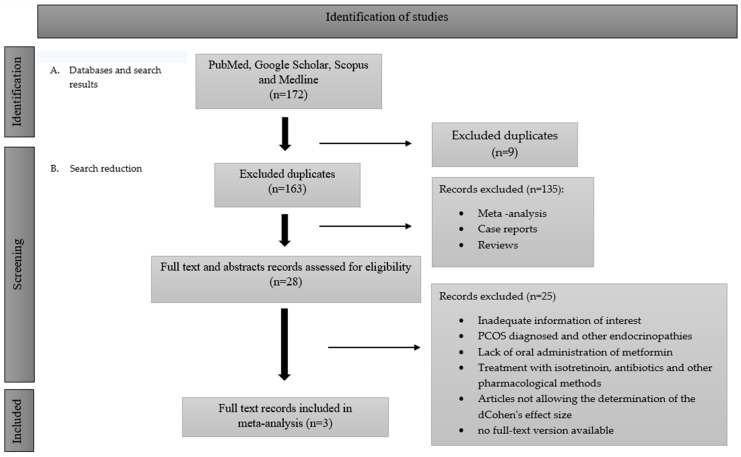
Article selection and reduction.

The study selection process is performed in Figure 3.

At the very beginning, after analyzing the title and keywords, articles similar in subject matter were selected. The next phase was performed to exclude the duplicates. Papers like meta-analyses, case reports, and reviews were also unnecessary. The next step involved a careful study of the abstracts of each article and the exclusion of those that were conducted in a manner other than oral metformin administration or were related to patients with PCOS or other endocrine diseases. After all three studies completed the above standards and were also performed with the same method of metformin administration (in monotherapy and not as an adjuvant therapy), they were included in our meta-analysis. The study protocol was registered in PROSPERO on 15 January 2024. ID CRD42024497391.

### 4.3. Statistical Analysis

Statistical analysis with accompanying figures was performed using PQStat v1.8.6 software. Heterogeneity was assessed using the I^2^ statistics and publication bias by visual inspection of the funnel plot. Meta-analysis was performed using Cohen’s d coefficient, which is defined as the difference between two means divided by the standard deviation of the data. It indicates how much the mean changes relative to the standard change. The value of the Pearson correlation coefficient required for the meta-analysis of related means was taken as an average of 0.5 due to the lack of information in the original articles.

### 4.4. Risk of Bias

**Figure 4:**
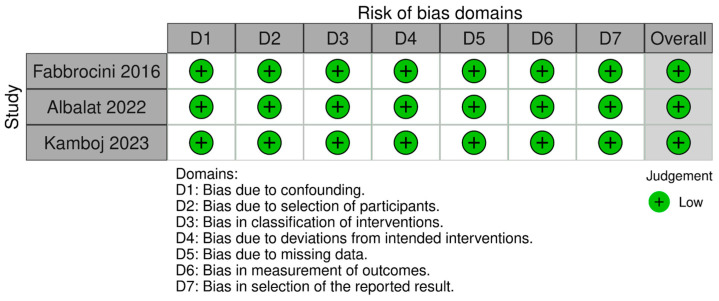
Bias assessment [29,30,31].

The bias assessment was performed using the ROBINS-I tool (https://www.riskofbias.info/welcome/robvis-visualization-tool; accessed date 23 May 2024). Two independent researchers were involved in this process. The sources utilized for assessing the risk of bias in the articles included in the meta-analysis comprised the articles themselves. All included in the meta-analysis articles were evaluated as having a low risk of bias. Results of the bias assessment are performed in Figure 4 [66].

### 4.5. GAGS Assessment 

To perform our meta-analysis, we selected studies with the same methods of acne assessment. All included studies contain data about acne evaluation in the GAGS. The GAGS divides acne occurrence location into six areas—forehead, right cheek, left cheek, nose, chin, chest, and upper back together. Each location is graded separately with 0–4 grades. Most severe acne lesion determines grading for a particular area and then it is multiplied by a factor assigned for this specific location. The global score is the result of the summation of individual scores. Depending on the obtained result, the GAGS defines acne as mild (1–18), moderate (19–30), severe (31–38), and very severe (>39) [67]. 

## 5. Conclusions

Our meta-analysis indicated a statistically significant improvement in acne grading after metformin treatment. It might be an appropriate direction for acne treatment in patients with resistance to standard therapies. We indicate the importance of finding the cause of acne and its relationship with other underlying hormonal or metabolic disorders, especially in acne resistant to symptomatic therapies. Further searches for causal therapies in acne treatment may contribute to improving patients’ QoL and reducing the occurrence of this condition. It is important to notice the safety of metformin therapy and consider its use in patients who do not tolerate other therapies well. However, it is still essential to conduct more adequate studies, which will be performed with a precise selection of control and study groups in terms of acne severity, duration of therapy, and the same method of metformin administration in acne treatment in monotherapy as well as adjuvant therapy.

## Figures and Tables

**Table 1:** Data collected from selected articles.

Author	Group	n	Age (±SD)	BMI (±SD)	Sex	Methods of Treatments	GAGS Pre-Treatment (±SD)	GAGS after Treatment (±SD)	*p*-Value
Fabbrocini 2016 [29]	Study	10	19.5	24.8 (±3)	10 M	Receive 500 mg metformin twice daily with a hypocaloric diet and symptomatic anti-acne treatment	25.1 (±8.9)	14.1 (±10.4)	*p* < 0.03
Albalat 2022 [30]	Study	50	18.7 (±3.1)	23.5 (±1.8)	33 F 17 M	Receive 500 mg metformin twice daily	25.2 (±6.8)	13.6 (±4.5)	*p* = 0.001
Kamboj 2023 [31]	Study	30	-	-	-	Receive 1000 mg of metformin	19.8 (±5.4)	13 (±5.79)	*p* < 0.001

**Table 2:** Effect of metformin therapy on Gags—the results of a meta-analysis.

Study Name	n	Cohen’s d	SE	−95% CI	+95% CI	Z-Value	*p*-Value	Weight (%)
Fabbrocini 2016 [29]	10	1.129706459	0.404736747	0.336437012	1.922975906	2.791212973	0.005251091	24.566%
Albalat 2022 [30]	50	1.936293823	0.239775599	1.466342285	2.406245361	8.075441501	<0.000000001	37.767%
Kamboj 2023 [31]	30	1.217018926	0.240871164	0.74492012	1.689117732	5.052572113	0.000000436	37.667%
Combined	90	1.467217174	0.273362471	0.931436576	2.002997772	5.36729555	0.00000008	

## Data Availability

The data presented in this study are available on request from the corresponding author.

## Abbreviations

IR	Insulin Resistance
GAGS	Global Acne Grading System
PCOS	Polycystic Ovary Syndrome
QoL	Quality of Life
IGF-1	Insulin-like Growth Factor 1
IGF-binding protein	Insulin-like Growth Factor Binding Protein
mTORC1	Mammalian Target of Rapamycin Complex 1
SHBG	Sex-hormone Binding Protein
AMPK	AMP-activated protein kinase
BMI	Body Mass Index
HOMA-IR	Homeostatic Model Assessment of Insulin Resistance
HIF-1α	Hypoxia-inducible Factor 1-α
IGA	Investigator’s Global Assessment
CADI	Cardiff Acne Disability Index
TLC	Total Lesions Count
DLQI	Dermatology Life Quality Index
SPF	Sun Protection Factor
PRISMA	Preferred Reporting Items for Systematic Reviews and Meta-Analyses
